# Evaluation of correlations between serum and urinary vitamin D metabolites using LC–MS/MS

**DOI:** 10.1042/BSR20260223

**Published:** 2026-07-01

**Authors:** Shixing Wu, Nau Ishimine, Riho Shimizu, Harue Suzuki, Yoko Usami, Taichi Shimazaki, Koji Takahashi, Masaki Takiwaki, Kentaro Abe, Sota Yoshimura, Ryota Sakamoto, Rino Tsutsumi, Kazuo Nagasawa

**Affiliations:** 1 Department of Medical Sciences, Graduate School of Medicine, Science and Technology, Shinshu University, Matsumoto, Japan; 2 Department of Laboratory Medicine, Shinshu University Hospital, Matsumoto, Japan; 3 ME Business Operations, JEOL Ltd., Tokyo, Japan; 4 Department of Biotechnology and Life Science, Tokyo University of Agriculture and Technology, Tokyo, Japan

**Keywords:** DAP-PA, LC-MS/MS, urine, vitamin D, vitamin D metabolite ratio (VMR), vitamin D metabolites

## Abstract

Vitamin D (VD) is involved in bone health, immunity, cardiovascular function, and cancer prevention. Recent studies suggested the potential of 24,25-dihydroxyvitamin D_3_ [24,25(OH)_2_D_3_] and the VD metabolite ratio (VMR = [24,25(OH)_2_D_3_/25(OH)D] × 100) as indicators of VD levels. An assessment of VD levels relies on serum measurements. The present study investigated the utility of urinary VD metabolites as a non-invasive alternative and their potential as surrogate markers for serum VD metabolites. We used residual serum and urine from the annual health check-ups of employees at Shinshu University Hospital, Japan (*n* = 506). Urine samples were mixed with β-glucuronidase. Serum and urine were processed via solid-phase extraction, followed by DAP-PA derivatization. LC–MS/MS was used to quantify serum and urinary 25(OH)D_3_, 25(OH)D_2_, 3-*epi*-25(OH)D_3_, and 24,25(OH)_2_D_3_ and urinary 23,25(OH)_2_D_3_. Serum whole parathyroid hormone (PTH) levels were measured, and urinary VD metabolites were adjusted for urinary creatinine. We also investigated whether serum and urinary VD metabolites correlated with PTH. The results showed that urinary 24,25(OH)_2_D_3_ and 23,25(OH)_2_D_3_ strongly correlated with serum 25(OH)D [25(OH)D_3_ + 25(OH)D_2_] (all ρ >0.7, *P*<0.001). Urinary 24,25(OH)_2_D_3_, 23,25(OH)_2_D_3_, and VMR had stronger inverse correlations with whole PTH (ρ = −0.297, −0.274, −0.308, respectively; all *P*<0.001), whereas serum 25(OH)D showed a weak correlation (ρ = −0.174, *P*<0.001). In conclusion, urinary VD metabolites, particularly 24,25(OH)_2_D_3_, 23,25(OH)_2_D_3_ and the VMR, correlated with serum values and PTH, suggesting their potential as non-invasive markers for the VD status.

## Introduction

Vitamin D plays a crucial role in bone metabolism and calcium homeostasis. Vitamin D deficiency has long been associated with rickets and has more recently been linked to impaired immune responses and increased risks of cancer and cardiovascular disease [1]. These physiological effects are largely mediated by 1,25-dihydroxyvitamin D [1,25(OH)_2_D], the active hormonal form of vitamin D. Its circulating levels are tightly regulated by parathyroid hormone (PTH) and fibroblast growth factor 23 (FGF23), which promote and suppress its synthesis, respectively. However, due to its short half-life in the circulation, 1,25(OH)_2_D is not regarded as a reliable indicator of the vitamin D status. Total 25-hydroxyvitamin D [25(OH)D]—the sum of 25-hydroxyvitamin D_3_ [25(OH)D_3_] and 25-hydroxyvitamin D_2_ [25(OH)D_2_]—is routinely measured as an indicator of the vitamin D status due to its longer half-life and greater stability in blood. Although 25(OH)D and 1,25(OH)_2_D have been the primary focus of clinical and research investigations, these markers alone do not fully capture the complexity of vitamin D metabolism. Therefore, recent studies have investigated additional metabolites, such as 3-*epi*-25-hydroxyvitamin D_3_ [3-*epi*-25(OH)D_3_], 24,25-dihydroxyvitamin D_3_ [24,25(OH)_2_D_3_], and 23,25-dihydroxyvitamin D_3_ [23,25(OH)_2_D_3_], to provide a more comprehensive assessment of vitamin D metabolic dynamics and function. Among these, the vitamin D metabolite ratio (VMR)—calculated as the ratio of 24,25(OH)_2_D_3_ to 25(OH)D—has emerged as a potentially more sensitive indicator of vitamin D catabolism. Wagner et al. [2] initially proposed the VMR, and subsequent studies confirmed that it more strongly correlated with PTH levels than total 25(OH)D [3,4].

Vitamin D is metabolized by members of the cytochrome P450 (CYP) enzyme family into various bioactive and inactive forms [5,6]. Vitamin D_3_, synthesized in the skin, and vitamin D_2_, obtained from dietary sources, are both 25-hydroxylated primarily by CYP2R1 and CYP27A1 in the liver, yielding 25(OH)D_3_ and 25(OH)D_2_, respectively. However, the amount of 25(OH)D_2_ in the circulation is generally very low. 25(OH)D_3_ then undergoes 1α-hydroxylation by CYP27B1 in the kidney to produce 1,25(OH)_2_D_3_, the hormonally active form of vitamin D. This active metabolite is then deactivated through 24-hydroxylation by CYP24A1, resulting in 1,24,25(OH)_3_D_3_ or conversion into a lactone form. CYP24A1 also inactivates 25(OH)D_3_ via 24-hydroxylation, producing 24,25(OH)_2_D_3_, a major catabolic product.

In addition to hydroxylated metabolites, some vitamin D compounds are present in conjugated forms in the circulation [7,8]. The urinary excretion of glucuronide-conjugated metabolites has been reported [9,10], whereas sulfate-conjugated forms have not been detected in urine [8].

Various analytical methodologies have been developed to measure vitamin D and its metabolites in blood, including enzyme-linked immunosorbent assays, radioimmunoassays, chemiluminescence immunoassays, gas chromatography-mass spectrometry, and liquid chromatography-tandem mass spectrometry (LC–MS/MS) [11]. While an immunoassay for urinary vitamin D metabolites has also been developed [12,13] it lacks the capacity to precisely quantify individual metabolites due to the widely varying affinities between different metabolites and the antibodies used. As the importance of distinguishing individual vitamin D metabolites has been increasingly recognized [14], LC–MS/MS has become the preferred method for accurate and specific quantification. However, LC–MS/MS presents challenges due to the inherently low ionization efficiency of native vitamin D compounds. To overcome this limitation and enhance sensitivity, various derivatization techniques have been developed [9,15–17]. Among these, 4-(4′-dimethylaminophenyl)-1,2,4-triazoline-3,5-dione (DAPTAD) has been used since 2013; however, its short shelf life and high moisture sensitivity limit its practical utility. In response to these issues, Seki et al. [14] developed a stabilized derivative, caged DAPTAD [14-(4-(dimethylamino)phenyl)-9-phenyl-9,10-dihydro-9,10-[1,2]epitriazoloanthracene-13,15-dione (DAP-PA)], in 2019, which enables extended storage and improved reliability. Furthermore, because the concentrations of urinary vitamin D metabolites are typically in the picogram-per-milliliter (pg/ml) range—markedly lower than those found in serum (ng/ml) [17]—highly sensitive and accurate quantification by LC–MS/MS is essential for a urinalysis.

While vitamin D metabolites in serum have been extensively studied and are widely used as clinical indicators of the vitamin D status, the significance of urinary vitamin D metabolites has yet to be examined in detail. Previous studies mostly focused on total serum 25(OH)D or the active form, 1,25(OH)_2_D, overlooking the dynamic aspects of vitamin D metabolism that may be reflected in urinary excretion profiles. This gap is particularly notable given that urine collection is non-invasive, low-risk, and potentially suitable for frequent monitoring in both clinical and epidemiological contexts. However, the clinical application of a urinary vitamin D metabolite analysis has been hindered by their extremely low concentrations (typically in the pg/ml range) and the absence of standardized, highly sensitive analytical protocols. To date, only a limited number of studies—such as one involving pregnant women [18]—have investigated the potential relationships between urinary vitamin D metabolites and physiological or pathological states. Therefore, it remains unclear whether urinary metabolites serve as reliable proxies for the vitamin D status or metabolic activity. Addressing this knowledge gap is essential for investigating whether urine-based assays complement or, under specific clinical conditions, potentially substitute for serum-based measurements.

Based on these findings, the present study investigated the potential utility of urinary vitamin D metabolites as non-invasive indicators of the vitamin D status by examining their quantitative relationships with serum metabolites and various blood biomarkers.

## Materials and methods

### Serum and urine samples

We herein used residual blood and urine samples collected during the 2024 annual health check-ups of employees at Shinshu University Hospital (3-1-1 Asahi, Matsumoto 3908621, Japan, latitude 36°N). No additional sample collection was performed. Samples from individuals who declined to participate were excluded from the analysis. All samples were stored at −80°C until analyzed.

Given that the present study used only residual specimens obtained during annual health check-ups, obtaining written informed consent individually from all participants was considered impractical. Instead, information regarding the use of samples for research purposes was provided at the time of health check-ups through posted notices and verbal explanation, and participants were given the opportunity to opt out.

This research has been carried out in accordance with the World Medical Association Declaration of Helsinki. The study protocol, including the opt-out consent procedure, was approved by the Ethics Committee of the Faculty of Medicine, Shinshu University (No. 6092), and the requirement for written informed consent was waived by the board.

### Observation and examination items

Information on sex, age, and body mass index was obtained from check-up interview sheets. Blood biochemical tests were performed to measure aspartate aminotransferase (AST), alanine aminotransferase (ALT), gamma-glutamyl transferase (γ-GT), total bilirubin, albumin, urea nitrogen, creatinine, whole PTH, vitamin D metabolites [25(OH)D_3_, 25(OH)D_2_, 3*-epi-*25(OH)D_3_, 24,25(OH)_2_D_3_, and 1,25(OH)_2_D_3_], and bone turnover markers [osteocalcin, type I procollagen N-terminal propeptide (total P1NP), and type I collagen cross-linked C-telopeptide (β-CTx)]. Urinary tests included measurements of creatinine and vitamin D metabolites [25(OH)D_3_, 25(OH)D_2_, 3*-epi-*25(OH)D_3_, 24,25(OH)_2_D_3_, and 23,25(OH)_2_D_3_]. 1,25(OH)_2_D_3_ was previously reported to be undetectable in urine [17].

Measurements of AST, ALT, γ-GT, total bilirubin, albumin, urea nitrogen, creatinine, and urinary creatinine were conducted at the Department of Laboratory Medicine, Shinshu University Hospital, which is accredited under ISO 15189 for medical laboratory quality and competence. All other biochemical and urinary analyses were performed at the same laboratory.

### Reagents for measuring vitamin D metabolites

The JeoQuant™ Kit for Vitamin D Metabolites (JEOL Ltd., Tokyo, Japan) was used to quantify 25(OH)D_3_, 25(OH)D_2_, 3*-epi-*25(OH)D_3_, and 24,25(OH)_2_D_3_. The accuracy of the calibration was confirmed using human serum reference materials provided by the National Institute of Standards and Technology, which further supported matrix-appropriate quantification. The 1,25(OH)_2_-Vitamin D_3_/D_2_ ImmuTube^®^ LC–MS/MS extraction kit (Immundiagnostik AG, Germany) was employed to measure 1,25(OH)_2_D_3_ [19]. Reagents including distilled water, acetonitrile, formic acid, methanol (LC–MS grade), 1 mol/l ammonium formate solution (HPLC grade), hexane, and ethyl acetate (special grade) were purchased from FUJIFILM Wako Pure Chemical Corporation (Osaka, Japan). Calibrators and internal standard (IS) solutions were prepared in-house by adjusting the concentrations of the JeoQuant™ calibrators and IS solutions to suit urinary vitamin D metabolite quantification. In addition, synthesized standards of 23,25-dihydroxyvitamin D_3_ [23,25(OH)_2_D_3_] and its deuterated analog 23,25(OH)_2_D_3_-*d*_2_ were incorporated. The concentrations of calibrators were as follows: 6.25, 12.5, 25, 50, 100, 200, 400, and 800 pg/ml for 25(OH)D_3_, 24,25(OH)_2_D_3_, and 23,25(OH)_2_D_3_; 3.125, 6.25, 12.5, 25, 50, 100, 200, and 400 pg/ml for 3-*epi*-25(OH)D_3_ and 25(OH)D_2_. The IS solution contained the following isotopically labeled compounds: 25(OH)D_3_-^13^C_5_, 25(OH)D_2_-^13^C_3_, 24,25(OH)_2_D_3_-^13^C_3_, and 23,25(OH)_2_D_2_-*d*_2_ at 1 ng/ml; 3*-epi-*25(OH)D_3_-^13^C_5_ at 0.1 ng/ml. All calibrators and IS solutions were prepared in-house. 23,25(OH)_2_D_3_ and 23,25(OH)_2_D_3_-*d*_2_ compounds were synthesized for the present study [20].

### Sample preparation for measuring serum vitamin D metabolites

Briefly, 50 μl of serum samples or standards were mixed with 250 μl of IS solution containing 25(OH)D_3_-^13^C_5_, 25(OH)D_2_-^13^C_3_, and 3*-epi-*25(OH)D_3_-^13^C_5_, 24,25(OH)_2_D_3_-^13^C_3._ The mixture was loaded onto a solid–liquid extraction (SLE) plate (ISOLUTE SLE+; Biotage, Uppsala, Sweden) and incubated at room temperature for 5 min. Vitamin D metabolites were then eluted three times with 600 μl of ethyl acetate/hexane (50/50, v/v), and the combined eluates were evaporated at 50°C using a positive pressure manifold (Biotage^®^ PRESSURE + 96; Biotage, Uppsala, Sweden). The dried residue was derivatized with DAP-PA as previously described [14]. Derivatization was performed by pre-heating at 80°C for 15 min, followed by at room temperature for an additional 15 min. After a second evaporation at 50°C under nitrogen gas, the residue was reconstituted in 60 μl of 50% acetonitrile (v/v), and 5 μl was injected into the LC–MS/MS system. The method was validated by Seki et al. [14].

To measure 1,25(OH)_2_D_3_, 100 μl of serum or standards was mixed with 200 μl of IS solution (1,25(OH)_2_D_3_-^13^C_3_) added to ImmuTubes^®^. The tubes were incubated at room temperature for 1 h using an overhead rotator. Following the incubation, the contents were centrifuged, washed, and eluted using reagents provided in the kit. The eluate was dried and derivatized with DAP-PA as described above. The final residue was reconstituted in 50 μl of 50% acetonitrile (v/v), and 20 μl was injected into the LC–MS/MS system. The method was validated by Ishige et al. [19].

### Validation of enzymatic hydrolysis conditions for the quantification of glucuronidated vitamin D metabolites in urine

#### Synthesis of glucuronidated vitamin D metabolites

**Figure 1:**
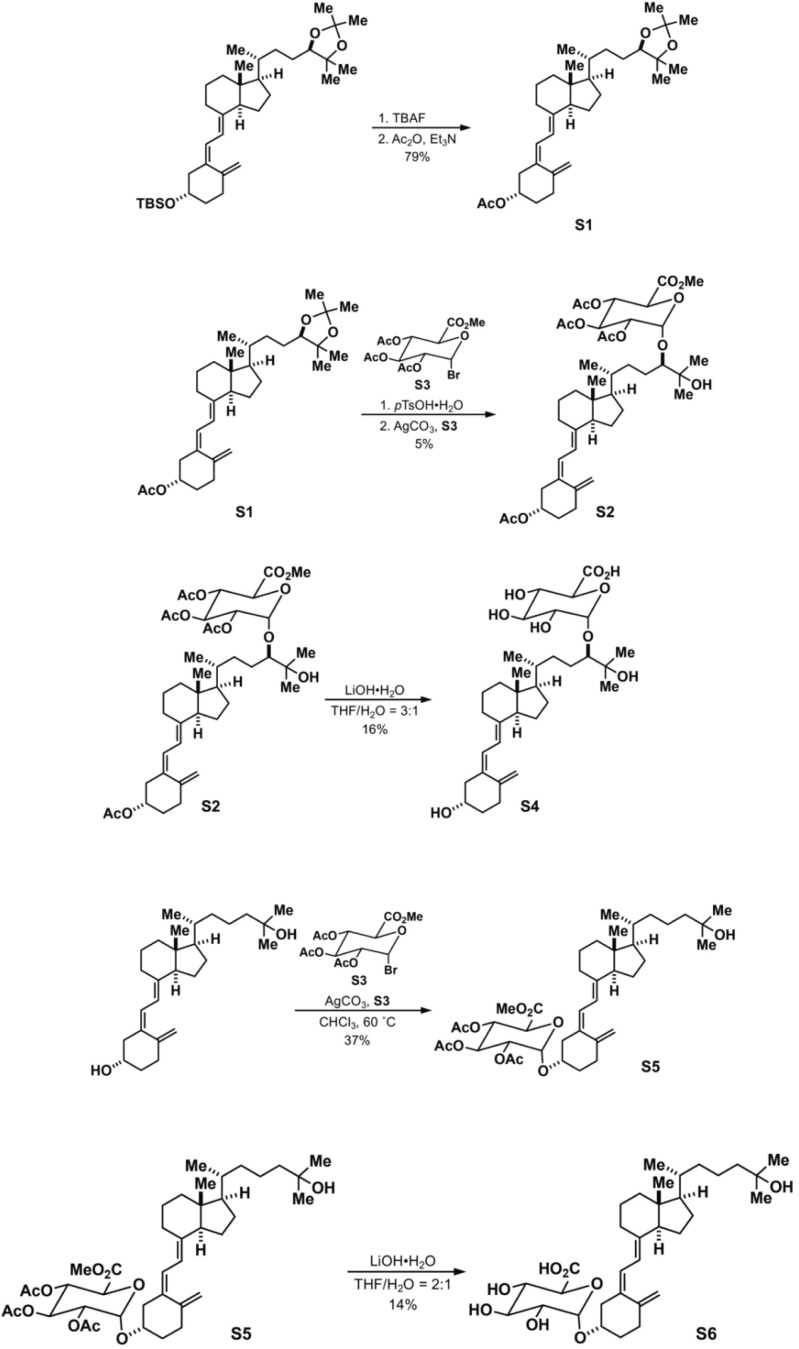
Synthesis of glucuronidated vitamin D metabolites The synthetic route of glucuronidated vitamin D metabolites for validating enzymatic hydrolysis conditions is shown.

Flash chromatography was performed using silica gel 60 (spherical, particle size 0.040–0.100 mm. Kanto Co., Inc., Japan). Preparative-TLC (PLC) was conducted using PLC Silica gel 60 F_254_ (0.5 mm, Merck Ltd., Germany). ^1^H NMR spectra were recorded on JEOL JNM-AL300 (300 MHz), JEOL JNM-ECX 400 (400 MHz), and JEOL JNM-ECA 500 (500 MHz). Chemical shifts in CDCl_3_, CD_3_OD, and DMSO-*d*_6_ were reported in scales relative to CDCl_3_ (7.26 ppm), CD_3_OD (3.31 ppm), and DMSO-*d*_6_ (2.50 ppm), respectively. The synthetic route is shown in Figure 1. The chemical structure of the derivatization reagent has been described in detail by Seki et al. [14]; therefore, readers are referred to the original publication for structural information.

##### (*S*,*Z*)-3-(2-((1*R*,3a*S*,7a*R*,*E*)-7a-Methyl-1-((*R*)-4-((*R*)-2,2,5,5-tetramethyl-1,3-dioxolan-4-yl)butan-2-yl)octahydro-4*H*-inden-4-ylidene)ethylidene)-4-methylenecyclohexyl acetate (S1)

Tetrabutylammonium fluoride (1 M solution in tetrahydrofuran (THF), 0.59 ml, 0.59 mmol) was added to a solution of the coupling product [20] (113 mg, 0.198 mmol) in THF (2.0 ml) at room temperature, and the mixture was stirred at the same temperature for 5 h. Saturated NH_4_Cl aq. was added to the reaction mixture and the resulting mixture was extracted with ethyl acetate three times. The combined extracts were washed with brine, dried over MgSO_4_, filtered, and concentrated *in vacuo*. The residue was purified by column chromatography on silica gel (*n*-hexane/ethyl acetate = 4:1) to give alcohol (81 mg, 90%). Et_3_N (0.15 ml, 1.07 mmol) and acetic anhydride (83 μl, 0.888 mmol) were added to a solution of alcohol (81 mg, 0.178 mmol) in CH_2_Cl_2_ (1.8 ml) at 0°C, and the mixture was stirred at room temperature for 30 min. The reaction mixture was quenched with H_2_O, and the resulting mixture was extracted with CH_2_Cl_2_ three times. The extracts were dried over MgSO_4_, filtered, and concentrated *in vacuo*. The residue was purified by column chromatography on silica gel (*n*-hexane/ethyl acetate = 20:1) to give acetate **S1** (77.9 mg, 88%) (2 steps 79%) as a colorless oil. **S1**: ^1^H NMR (300 MHz, CDCl_3_) δ 6.21 (d, *J* = 11.5 Hz, 1H), 6.02 (d, *J* = 11.5 Hz, 1H), 5.05 (s, 1H), 4.93 (m, 1H), 4.83 (s, 1H), 3.64 (dd, *J* = 8.6, 3.5 Hz, 1H), 2.81 (dd, *J* = 12.4, 3.0 Hz, 1H), 2.56 (dd, *J* = 13.2, 3.6 Hz, 1H), 2.44-2.32 (m, 2H), 2.23-2.13 (m, 1H), 2.04 (s, 3H), 1.98-1.44 (m, 22H), 1.41 (s, 2H), 1.33 (s, 3H), 1.25 (s, 3H), 0.95 (d, *J* = 8.4 Hz, 3H), 0.55 (s, 3H).

##### (2*S*,3*R*,4*S*,5*S*,6*S*)-2-(((3*R*,6*R*)-6-((1*R*,3a*S*,7a*R*,*E*)-4-((*Z*)-2-((*S*)-5-Acetoxy-2-methylenecyclohexylidene)ethylidene)-7a-methyloctahydro-1*H*-inden-1-yl)-2-hydroxy-2-methylheptan-3-yl)oxy)-6-(methoxycarbonyl)tetrahydro-2*H*-pyran-3,4,5-triyl triacetate (S2)

*p*-Toluenesulfonic acid monohydrate (89.0 mg, 0.468 mmol) was added to a solution of acetate **S1** (77.9 mg, 0.156 mmol) in MeOH (2.6 ml) at room temperature, and the mixture was stirred at the same temperature for 1.5 h. The reaction mixture was quenched with saturated NaHCO_3_ aq. and the resulting mixture was extracted with ethyl acetate three times. The combined layer was washed with brine, dried over MgSO_4_, filtered, and concentrated *in vacuo*. The residue was purified by column chromatography on silica gel (*n*-hexane/ethyl acetate 8:1) to give diol (20 mg, 28%). Ag_2_CO_3_ (18.1 mg, 0.0668 mmol) and acetobromo-α-_D_-glucuronic acid methyl ester **S3** (25.2 mg, 0.0635 mmol) were added to a solution of the diol (5.8 mg, 0.0127 mmol) in CHCl_3_ (0.13 ml) at room temperature under an argon atmosphere, and the resulting mixture was heated at 60°C. After stirring for 3.5 h, the reaction mixture was cooled to room temperature and quenched with saturated NaHCO_3_ aq., and the resulting mixture was extracted with ethyl acetate three times. The combined extracts were washed with brine, dried over MgSO_4_, filtered, and concentrated *in vacuo*. The residue was purified by column chromatography on silica gel (CHCl_3_/ethyl acetate = 5:1, 1% Et_3_N) to give acetate **S2** (1.8 mg, 18%) (2 steps 5%) as a colorless oil. **S2**: ^1^H NMR (300 MHz, CDCl_3_) δ 6.21 (d, *J* = 11.2 Hz, 1H), 6.03 (d, *J* = 11.2 Hz, 1H), 5.24 (m, 3H), 5.07 (s, 1H), 4.94 (m, 1H), 4.83 (s, 1H), 4.64 (d, *J* = 8.4 Hz, 1H), 4.05 (d, *J* = 9.2 Hz, 1H), 3.75 (s, 3H), 3.49 (s, 1H), 3.38 (d, *J* = 9.2 Hz, 1H), 2.81 (d, *J* = 8.0 Hz, 1H), 2.57 (d, *J* = 13.6 Hz, 1H), 2.39 (m, 2H), 2.07-1.12 (m, 39H), 0.91 (d, *J* = 6.4 Hz, 3H), 0.54 (s, 3H).

##### (2*S*,3*S*,4*S*,5*R*,6*S*)-3,4,5-Trihydroxy-6-(((3*R*,6*R*)-2-hydroxy-6-((1*R*,3a*S*,7a*R*,*E*)-4-((*Z*)-2-((*S*)-5-hydroxy-2-methylenecyclohexylidene)ethylidene)-7a-methyloctahydro-1*H*-inden-1-yl)-2-methylheptan-3-yl)oxy)tetrahydro-2*H*-pyran-2-carboxylic acid (24-glc-25(OH)D_3_, S4)

LiOH•H_2_O (1.3 mg, 0.0310 mmol) was added to a solution of acetate **S2** (0.8 mg, 0.00103 mmol) in THF (22 μl) and H_2_O (7 μl) at room temperature, and the mixture was stirred at the same temperature for 24 h. The reaction mixture was concentrated *in vacuo*, and the residue was subjected to PLC (CHCl_3_/MeOH/H_2_O = 70:30:4). The zone corresponding to *Rf ca.* 0.4 was collected and extracted (CHCl_3_/MeOH = 9:1) to give 24-glc-25(OH)D_3_ **S4** (0.1 mg, 16%) as a colorless oil. **S4**: ^1^H NMR (500 MHz, CD_3_OD) δ 6.20 (d, *J* = 10.5 Hz, 1H), 6.00 (d, *J* = 10.5 Hz, 1H), 5.05 (s, 1H), 4.71 (s, 1H), 4.31 (d, *J* = 8.0 Hz, 1H), 3.92 (m, 1H), 3.72 (m, 1H), 3.85 (d, *J* = 12.5 Hz, 1H), 2.50 (d, *J* = 12.5 Hz, 1H), 0.92 (d, *J* = 6.0 Hz, 3H), 0.53 (s, 3H).

##### (2*S*,3*R*,4*S*,5*S*,6*S*)-2-(((*S*,*Z*)-3-(2-((1*R*,3a*S*,7a*R*,*E*)-1-((*R*)-6-hydroxy-6-methylheptan-2-yl)-7a-methyloctahydro-4*H*-inden-4-ylidene)ethylidene)-4-methylenecyclohexyl)oxy)-6-(methoxycarbonyl)tetrahydro-2*H*-pyran-3,4,5-triyl triacetate (S5)

Ag_2_CO_3_ (36.2 mg, 0.131 mmol) and acetobromo-α-_D_-glucuronic acid methyl ester (49.7 mg, 0.125 mmol) were added to a solution of 25(OH)D_3_ (10 mg, 0.025 mmol) in CHCl_3_ (0.25 ml) at room temperature under an argon atmosphere and the reaction mixture was heated at 60°C for 3.5 h. The reaction mixture was quenched with saturated NaHCO_3_ aq. and the resulting mixture was extracted with ethyl acetate three times. The combined extracts were washed with brine, dried over MgSO_4_, filtered, and concentrated *in vacuo*. The residue was purified by column chromatography on silica gel (CHCl_3_/ethyl acetate = 5:1, 1% Et_3_N) to give acetate **S5** (6.6 mg, 37%) as a colorless oil. **S5**: ^1^H NMR (300 MHz, CDCl_3_) δ 6.16 (d, *J* = 11.4 Hz, 1H), 6.00 (d, *J* = 11.4 Hz, 1H), 5.23 (m, 3H), 5.03 (s, 1H), 4.98 (m, 1H), 4.80 (s, 1H), 4.68 (d, *J* = 8.8 Hz, 1H), 4.02 (d, *J* = 8.8 Hz, 1H), 3.95 (m, 1H), 3.75 (s, 3H), 3.64 (dd, *J* = 8.6, 3.5 Hz, 1H), 2.81 (dd, *J* = 8.0, 3.8 Hz, 1H), 2.47 (dd, *J* = 8.0, 3.8 Hz, 1H), 2.37 (m, 1H), 2.30 (m, 1H), 2.11 (d, *J* = 5.2 Hz, 3H), 2.02-1.21 (m, 31H), 0.93 (d, *J* = 6.4 Hz, 3H), 0.53 (s, 3H).

##### (2*S*,3*S*,4*S*,5*R*,6*S*)-3,4,5-trihydroxy-6-(((*S*,*Z*)-3-(2-((1*R*,3a*S*,7a*R*,*E*)-1-((*R*)-6-hydroxy-6-methylheptan-2-yl)-7a-methyloctahydro-4*H*-inden-4-ylidene)ethylidene)-4-methylenecyclohexyl)oxy)tetrahydro-2*H*-pyran-2-carboxylic acid (3-glc-25(OH)D_3_, S6)

LiOH•H_2_O (4.7 mg, 0.1120 mmol) was added to a solution of acetate **S5** (0.9 mg, 0.00126 mmol) in THF (72 μl) and H_2_O (36 μl) at room temperature, and the mixture was stirred at the same temperature for 24 h. The reaction mixture was concentrated *in vacuo*, and the residue was subjected to PLC (CHCl_3_/MeOH/H_2_O = 70:30:4). The zone corresponding to *Rf ca.* 0.4 was collected and extracted (CHCl_3_/MeOH = 9:1) to yield 3-glc-25(OH)D_3_ **S6** (0.1 mg, 14%) as a colorless oil. **S6**: 1H NMR (400 MHz, DMSO-*d*_6_) δ 6.24 (d, *J* = 11.4 Hz, 1H), 5.95 (d, *J* = 11.4 Hz, 1H), 5.05 (s, 1H), 4.80 (s, 1H), 4.74 (s, 1H), 4.70 (s, 1H), 4.23 (s, *J* = 7.6 Hz), 4.04 (s, 1H), 3.77 (brs, 1H), 3.60 (brs, 1H), 3.51 (s, 1H), 3.38 (s, 1H), 3.16 (d, *J* = 9.8 Hz, 1H), 3.09 (m, 1H), 3.02 (d, *J* = 9.8 Hz, 1H), 2.88 (m, 1H), 2.81 (d, *J* = 12.2 Hz, 1H), 2.64 (d, *J* = 13.2 Hz, 1H), 2.35 (m, 1H), 2.15-1.92 (m, 6H), 1.87-1.74 (m, 2H), 1.69-1.10 (m, 20H), 1.05 (s, 7H), 0.90 (d, *J* = 6.0 Hz, 3H), 0.48 (s, 3H).

#### Analytical method for glucuronidated vitamin D metabolites in urine using LC–MS/MS

##### Preparation of urine samples and synthesized standards for a glucuronidated vitamin D metabolite analysis

To evaluate the suitability of enzymatic hydrolysis conditions for the quantification of glucuronidated vitamin D metabolites in urine, we conducted a confirmation experiment using in-house synthesized standards of 24-glc-25(OH)D_3_ and 3-glc-25(OH)D_3_. These standards were spiked into pooled human urine to achieve a final concentration of 10 ng/ml for each metabolite, thereby preparing urine samples enriched with glucuronidated vitamin D metabolites.

Regarding enzymatic hydrolysis, 300 μl of prepared urine was incubated with 50 μl of β-glucuronidase solution (Sigma–Aldrich, G7396-1MU, 5000 Fishman units) at 37°C for 1 h. Post-incubation, 40 μl of 1% acetic acid was added to the sample, which was then loaded onto an Oasis PRiME HLB μElution plate (Waters, Milford, MA, USA). The plate was washed sequentially with 200 μl of distilled water and 200 μl of 20% methanol, followed by elution with 100 μl of methanol. The eluate was dried and derivatized using DAP-PA, and then reconstituted in 100 μl of 50% (v/v) acetonitrile. A 10-μl aliquot was injected into the LC–MS/MS system (Xevo TQ-XS).

##### LC–MS/MS analytical conditions for the detection of glucuronidated vitamin D metabolites

Pretreated samples were analyzed using an ACQUITY UPLC I-Class PLUS System coupled with a Xevo TQ-XS System (Waters, Massachusetts, U.S.). A CAPCELL CORE C18 column (2.7 μm, 2.1 mm I.D. × 75 mm, OSAKA SODA, Osaka, Japan) was used at a flow rate of 0.4 ml/min and maintained at a temperature of 40°C. The mobile phases consisted of methanol-10 mM ammonium formate (7:3, v/v) for phase A and methanol-10 mM ammonium formate (9:1, v/v) for phase B. The gradient program was as follows: 0–2.50 min, 0% B; 2.51–3.50 min, 100% B; 3.51–4.50 min, 0% B. Electrospray ionization (ESI) was performed in the positive-ion mode with a capillary voltage of 1 kV, desolvation temperature of 550°C, desolvation gas flow of 1200 L/h, and source temperature of 150°C. Selected reaction monitoring (SRM) transitions (*m/z*) were optimized for the derivatized metabolites: *m/z* 795.14 → 341.1 (CE: 50 eV) for 3-glc-25(OH)D_3_ and *m/z* 811.2 → 341.1 (CE: 50 eV) for 24-glc-25(OH)D_3_.

### Sample and standard preparation for measuring urinary vitamin D metabolites

To hydrolyze glucuronidated vitamin D metabolites, 300 μl of urine samples were mixed with 50 μl of β-glucuronidase solution and incubated under optimized enzymatic conditions as described above. Following the enzymatic treatment, 300 μl of the hydrolyzed sample or standard was combined with 50 μl of IS solution and applied to an SLE plate. Subsequent steps—incubation, elution, evaporation, derivatization with DAP-PA, and reconstitution—were performed as described in the serum sample preparation section. A 20-μl aliquot of the final reconstituted solution was injected into the LC–MS/MS system.

### LC–MS/MS conditions of serum and urinary vitamin D metabolites

A sample analysis was conducted using an ACQUITY UPLC I-Class PLUS System coupled with a Xevo TQ-XS System. Chromatographic separation was achieved on a CAPCELL CORE C18 column using a binary mobile phase system.

In serum analyses, water (eluent A) and acetonitrile (eluent B), both containing 0.1% (v/v) formic acid, were used. The flow rate and total run time were set at 0.5 ml/min and 6 min, respectively. Gradient conditions were as follows: initial (70% of eluent A), 0–1.00 min (linear gradient to 56% eluent B), 1.00–3.00 min (56% eluent B), 3.00–3.50 min (linear gradient to 65% eluent B), 3.50–3.67 (linear gradient to 90% eluent B), 3.67–4.67 min (90% eluent B), 4.67–4.68 (linear gradient to 70% eluent A), and 4.68–6.00 min (70% eluent A for re-equilibration of the column).

In urine analyses, methanol/10 mmol/l ammonium formate solution (70/30, v/v) (eluent A) and methanol/10 mmol/l ammonium formate solution (90/10, v/v) (eluent B) was used. The flow rate and total run time were set at 0.4 ml/min and 12 min, respectively. Gradient conditions were as follows: 0–5.00 min (100% eluent A), 5.00–10.00 min (linear gradient to 40% eluent B), 10.00–10.01 min (linear gradient to 100% eluent B), 10.01–11.00 (100% eluent B), 11.00–11.01 min (linear gradient to 100% eluent A), and 11.01–12.00 min (100% eluent A for the re-equilibration of the column).

In serum and urine analyses, the column and autosampler temperatures were maintained at 40°C and 5°C, respectively. Samples were ionized using the ESI+ source under a capillary voltage 1.0 kV, desolvation temperature 550°C, and desolvation gas flow 1,200 L/h. Selected SRM was used for quantification. SRM transitions (*m/z*) were monitored as follows: 25(OH)D_3_ *m/z* 619.4 → 341.1; 25(OH)D_3_-IS *m/z* 624.4 → 341.1; 25(OH)D_2_ *m/z* 631.4 → 341.1; 25(OH)D_2_-IS *m/z* 634.1 → 341.1; 3-*epi*-25(OH)D_3_ *m/z* 619.4 → 341.1; 3-*epi*-25(OH)D_3_-IS *m/z* 624.4 (serum) 624.1 (urine) → 341.1; 24,25(OH)_2_D_3_ *m/z* 635.4 → 341.1; 24,25(OH)_2_D_3_-IS *m/z* 638.4 → 341.1; 23,25(OH)_2_D_3_ *m/z* 635.4 → 341.1; 23,25(OH)_2_D_3_-IS *m/z* 638.4 → 344.1; 1,25(OH)_2_D *m/z* 635.4 → 357.2; and 1,25(OH)_2_D-IS *m/z* 638.4 → 357.2.

MassLynx software (version 4.2, Waters) was used for instrument control and TargetLynx Browser (Waters) for data processing and quantification.

### Validation of the LC–MS/MS assay for serum and urinary vitamin D metabolites

Between-run precision was assessed by analyzing two concentration levels of serum control samples in quintuplicate and a single level of urine control samples in quadruplicate. Between-day precision was evaluated by measuring these control samples across six independent working days. Pooled urine samples obtained from the annual health check-up were used for the validation of the urinary assay. A coefficient of variation (CV) threshold of 10% was applied as the acceptance criterion. Dilution linearity was assessed using a serial dilution of pooled urine with distilled water. Spike-and-recovery was evaluated by spiking a pooled urine sample with distilled water or with calibrators at two concentrations (100 and 800 pg/ml), mixed at a volume ratio of 1.6 and 0.4 ml, respectively.

### Measurement of other biochemical parameters

Serum whole PTH, osteocalcin, total P1NP, and β-CTx were measured by an electrochemiluminescence immunoassay using the cobas pro e801 module (Roche Diagnostics).

Urinary creatinine concentrations were assessed using the BioMajesty™ ZERO series JCA-ZS050 (JEOL Ltd., Tokyo, Japan) and Cygnus Auto CRE (Shino-Test Corporation, Tokyo, Japan). Serum AST, ALT, urea nitrogen, creatinine, γ-GT, albumin, and total bilirubin were analyzed using the BioMajesty™ Series JCA-BM8020 (JEOL Ltd., Tokyo, Japan). The following reagent kits were employed: Quick Auto Neo AST JS, Quick Auto Neo ALT JS, Cygnus Auto UN, and Cygnus Auto CRE (Shino-Test Corporation, Tokyo, Japan); CicaLiquid γ-GT J and CicaLiquid ALB (KANTO CHEMICAL Co., Inc., Tokyo, Japan); and Nescauto VL T-BIL (Alfresa Pharma Corporation, Osaka, Japan).

### Data and statistical analyses

The VMR was used as an indicator of the vitamin D status, as previously described [2]. It was defined as the following formula.

$$\text{VMR}=\left[\frac{24,25{\left(\text{OH}\right)}_{2}{\text{D}}_{3}}{25\left(\text{OH}\right)\text{D}}\right]\times 100.$$

Differences in biochemical parameters between sexes were analyzed using the Mann–Whitney U test. Relationships among biomarkers were evaluated using Spearman’s rank correlation coefficient (ρ), and differences between correlation coefficients were evaluated using Steiger’s test. Subjects were categorized into quartiles according to individual vitamin D metabolite levels, and differences in PTH levels among quartile groups were analyzed using the Steel–Dwass test. Statistical significance was set at *P*<0.05. The study was statistically powered (80%) to detect an absolute difference of 0.2 in Spearman’s rank correlation coefficient, requiring a minimum sample size of 190 participants. Similarly, for the Steel–Dwass test, a minimum of 124 participants per quartile group was required to detect estimated differences among the four groups.

Urinary vitamin D metabolites were normalized to urinary creatinine concentrations using the following formula.

$$\text{Adjusted value}=\left[\frac{\text{metabolite concentration}}{\text{urinary creatinine}}\right]\times 100.$$

All statistical analyses were conducted using EZR (Saitama Medical Center, Jichi Medical University, Saitama, Japan) [21], a graphical user interface for R (The R Foundation for Statistical Computing, Vienna, Austria).

## Results

### Assessment of enzymatic hydrolysis efficiency for glucuronidated vitamin D metabolites in urine

Prior to enzymatic hydrolysis, an LC–MS/MS analysis of urine samples revealed distinct peaks corresponding to 3-glc-25(OH)D_3_ and 24-glc-25(OH)D_3_ (Supplementary Figure S1). Following hydrolysis, these peaks were completely absent, indicating the successful cleavage of the glucuronide moieties. Concurrently, non-conjugated vitamin D metabolites were detected in the hydrolyzed samples. These results confirmed that the enzymatic digestion conditions employed resulted in the near-complete hydrolysis of glucuronidated vitamin D metabolites, validating the method for subsequent quantitative analysis processing.

### Validation of serum and urinary vitamin D metabolites measured by LC–MS/MS

Although comprehensive method validation was not performed, assay precision was evaluated to confirm the reliability of LC–MS/MS measurements. Regarding serum vitamin D metabolites, within-run and between-run CVs ranged from 0.8% to 5.2% and from 4.1% to 8.2%, respectively (Supplementary Table S1). In urine, CVs for 25(OH)D_3_, 24,25(OH)_2_D_3_, and 23,25(OH)_2_D_3_ ranged from 2.0% to 4.5% (within-run) and 2.7% to 9.7% (between-run) (Supplementary Table S2). In contrast, urinary 3-*epi*-25(OH)D_3_ exhibited high variability, and urinary 25(OH)D_2_ was barely detectable in pooled urine samples.

Consequently, further validation tests, including linearity and spike-and-recovery assessments, were conducted for urinary 25(OH)D_3_, 24,25(OH)_2_D_3_, and 23,25(OH)_2_D_3_. The linearity of calibration curves was satisfactory, with *r* values exceeding 0.998 (Supplementary Figure S2). Spike-and-recovery tests yielded acceptable recovery rates ranging from 88% to 112% (Supplementary Table S3), supporting the reliability of quantification for these urinary metabolites.

### Serum and urinary concentrations of vitamin D metabolites

A total of 506 individuals were enrolled (156 males and 350 females), with a median age of 41 years (interquartile range (IQR), 33-51 years). The serum and urinary concentrations of vitamin D metabolites, along with other biochemical parameters, are summarized in Table 1. Representative chromatograms and raw data obtained from a urine sample are shown in Supplementary Figure S3. Significant sex differences were observed in serum vitamin D metabolites, with males showing higher concentrations than females for all metabolites, except 25(OH)D_2_. In contrast, sex differences in urinary metabolites were less consistent. Urinary 25(OH)D_3_, 25(OH)D_2_, 25(OH)D, and 23,25(OH)_2_D_3_ significantly differed between the sexes, whereas 3-*epi*-25(OH)D_3_, 24,25(OH)_2_D_3_, and the VMR did not. Since urinary metabolite concentrations were normalized to creatinine, which itself differs between the sexes (higher in males), the apparent sex differences in urinary metabolites may have been attenuated. Notably, the urinary VMR, which includes creatinine in both the numerator and denominator, may be affected less by sex differences in urinary creatinine levels. The median serum 25(OH)D level in the overall cohort was 22.33 ng/ml. Based on Japanese guidelines for the vitamin D status—deficiency (<20 ng/ml), insufficiency (≥20 ng/ml and <30 ng/ml), and sufficiency (≥30 ng/ml) [22], 39.5% of participants were classified as deficient and 42.1% as insufficient. Therefore, 81.6% had a suboptimal vitamin D status, consistent with findings from a local Japanese population study by Miyamoto et al. [23]. While these criteria are widely used, their appropriateness remains under discussion [24,25].

**Table 1:** Descriptive statistics of the study population

Variables	Median (IQR)	Median male	Median female	*P*-value^2^
Age	41 (33–51)	35	44	<0.001
Body mass index (kg/m^2^)	21.1 (19.6–23.2)	22.7	20.5	<0.001
Serum				
AST (U/l)	17 (15–21)	18	17	0.025
ALT (U/l)	15 (11–21)	19	14	<0.001
γ-GT (U/l)	17 (13–26)	22	16	<0.001
Total bilirubin (mg/dl)	0.68 (0.52–0.89)	0.82	0.63	
(μmol/l)	11.63 (8.89–15.22)	14.02	10.77	<0.001
Albumin (g/dl)	4.4 (4.2–4.6)	4.6	4.3	<0.001
(g/l)	44 (42–46)	46	43	
Urea nitrogen (mg/dl)	12.8 (10.8–15.3)	13.6	12.4	<0.001
(mmol/l)	4.57 (3.86–5.46)	4.86	4.43	
Creatinine (mg/dl)	0.73 (0.63–0.86)	0.91	0.67	<0.001
(μmol/l)	64.5 (55.7–76.0)	80.4	59.2	
Whole PTH (pg/ml)	42.15 (33.73–52.30)	43.60	41.10	0.476
Osteocalcin (ng/ml)	16.9 (13.5–21.6)	18.3	15.7	<0.001
Total P1NP (ng/ml)	54.15 (41.30–70.25)	62.45	51.35	<0.001
β-CTx (ng/ml)	0.21 (0.14–0.33)	0.25	0.19	<0.001
25(OH)D_3_ (ng/ml)	21.65 (17.02–26.91)	24.46	20.30	<0.001
25(OH)D_2_ (ng/ml)	0.52 (0.37–0.77)	0.48	0.54	0.023
25(OH)D (ng/ml)^1^	22.33 (17.79–27.66)	25.12	20.98	<0.001
3*-epi-*25(OH)D_3_ (ng/ml)	0.76 (0.59–1.00)	0.92	0.69	<0.001
24,25(OH)_2_D_3_ (ng/ml)	1.03 (0.65–1.47)	1.23	0.92	<0.001
1,25(OH)_2_D_3_ (pg/ml)	42.40 (35.40–50.68)	43.55	41.36	0.125
VMR	4.53 (3.61–5.63)	4.97	4.34	<0.001
Urine				
Creatinine (mg/dl)	104.25 (58.75–157.18)	149.25	81.80	<0.001
(μmol/l]	9215.88 (5193.60–13894.98)	13193.96	7231.26	
25(OH)D_3_ (ng/g·Cre)	21.26 (15.54–29.12)	23.00	20.82	0.011
25(OH)D_2_ (ng/g·Cre)	1.98 (1.03–3.51)	1.35	2.44	0.043
25(OH)D (ng/g·Cre)^1^	21.39 (15.61–29.20)	23.45	20.85	0.015
3*-epi-*25(OH)D_3_ (ng/g·Cre)	5.18 (3.19–8.12)	4.92	5.19	0.903
24,25(OH)_2_D_3_ (ng/g·Cre)	75.85 (49.93–113.75)	80.92	71.37	0.136
23,25(OH)_2_D_3_ (ng/g·Cre)	71.20 (44.90–110.32)	80.27	67.84	0.013
VMR	351.14 (251.74–467.34)	341.21	360.12	0.361

IQR, interquartile range; AST, aspartate aminotransferase; ALT, alanine aminotransferase; γ-GT, gamma-glutamyl transferase; PTH, parathyroid hormone; Total P1NP, type I procollagen N-terminal propeptide; β-CTx, type I collagen cross-linked C-telopeptide; VMR, vitamin D metabolite ratio.

1 25(OH)D is a summation of 25(OH)D_3_ and 25(OH)D_2_.

2 The Mann-Whitney U test of sex differences, two-sided.

The distributions of individual serum and urinary vitamin D metabolites, as well as the VMR, are shown in Supplementary Figure S4. Herrmann et al. [3] defined a serum VMR <4% as indicative of a low vitamin D metabolite profile. In the present study, 170 participants (33.6%) met this criterion.

### Correlations between serum and urinary vitamin D metabolites measured by LC–MS/MS

**Figure 2:**
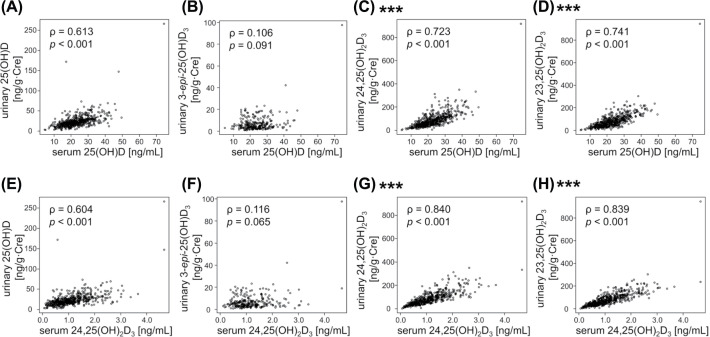
Correlations between serum and urinary vitamin D metabolites measured by LC–MS/MS Urinary metabolites were compared with serum 25-hydroxyvitamin D [25(OH)D] and 24,25-dihydroxyvitamin D_3_ [24,25(OH)_2_D_3_]. Spearman’s ρ was calculated, and Steiger’s test was applied between (**A**) and (**C**), (**A**) and (**D**), (**E**) and (**G**), and (**E**) and (**H**). The test was not performed for panels without correlations (**B,F**). ***P<0.001. Sample sizes: (**A,E**) 506; (**B,F**) 255; (**C,G**) 505; (**D,H**) 504.

All variables showed a non-normal distribution, as visually confirmed by histograms and quantile–quantile plots. Therefore, Spearman’s rank correlation was used to assess the relationships between serum and urinary vitamin D metabolites (Figure 2).

Strong positive correlations (ρ >0.6, *P*<0.001) were observed between serum 25(OH)D and urinary 24,25(OH)_2_D_3_ and 23,25(OH)_2_D_3_, as well as between serum 24,25(OH)_2_D_3_ and the same urinary metabolites. In contrast, urinary 25(OH)D and 3-*epi*-25(OH)D_3_ showed weaker or no correlations with serum 25(OH)D or 24,25(OH)_2_D_3_.

### Correlations between vitamin D metabolites and bone turnover markers

**Figure 3:**
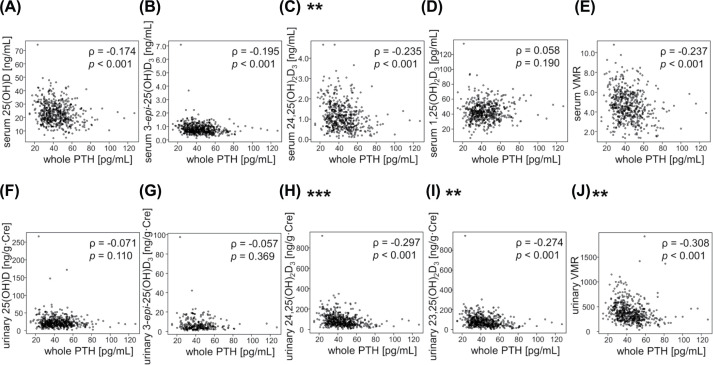
Correlations between vitamin D metabolites and whole PTH Serum and urinary metabolites were compared with PTH. Spearman’s ρ was calculated, and Steiger’s test was applied between (**A**) and others, respectively. **P<0.01, ***P<0.001. Sample sizes: (**A-F**) 506; (**G**) 255; (**H,J**) 505; (**I**) 504.

Consistent with previous studies [3,4,26], the present results showed that serum 25(OH)D, 24,25(OH)_2_D_3_, and the VMR weakly and inversely correlated with whole PTH levels. Notably, serum 24,25(OH)_2_D_3_ and VMR exhibited stronger negative associations with PTH than the conventional marker 25(OH)D_3_, although only the correlation with serum 24,25(OH)_2_D_3_ reached significance in Steiger’s test (Figure 3, serum).

**Figure 4:**
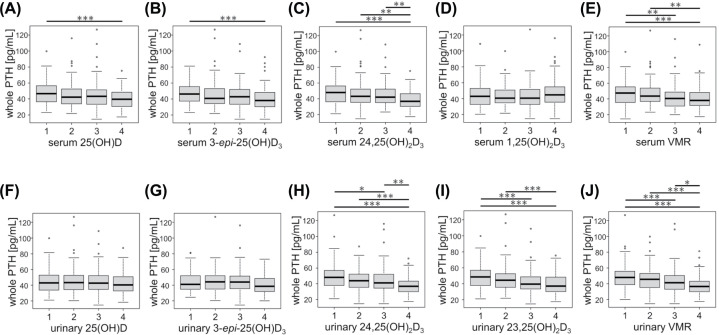
Whole PTH significantly differed among quartiles of vitamin D metabolites Serum and urinary metabolites were compared with PTH. The Steel-Dwass test was applied. *P<0.05, **P<0.01, ***P<0.001. Sample sizes: (**A-F**) 506; (**G**) 255; (**H,J**) 505; (**I**) 504.

Urinary vitamin D metabolites, particularly 24,25(OH)_2_D_3_, 23,25(OH)_2_D_3_, and the VMR, showed stronger inverse correlations with whole PTH than their serum counterparts (Figure 3, urine). These urinary markers also showed significant differences in whole PTH levels across quartiles (Figure 4, urine).

In contrast, serum 1,25(OH)_2_D_3_ did not correlate with whole PTH (Figures 3 and 4, serum). Similarly, no correlations were observed between serum or urinary vitamin D metabolites and bone turnover markers, including osteocalcin, total P1NP, and β-CTx (Table 2), which is consistent with previous findings [4]. However, the urinary VMR negatively correlated with bone turnover markers (Table 2).

**Table 2:** Relationships between vitamin D metabolites, VMR, and bone turnover markers

Variables	Osteocalcin		Total P1NP		β-CTx	
	ρ^2^	*P*-value^2^	ρ^2^	*P*-value^2^	ρ^2^	*P*-value^2^
Serum						
25(OH)D (ng/ml)^1^	0.035	0.438	0.040	0.367	0.038	0.400
3*-epi-*25(OH)D_3_ (ng/ml)	0.047	0.294	0.069	0.124	0.059	0.185
24,25(OH)_2_D_3_ (ng/ml)	0.017	0.702	0.016	0.722	0.028	0.524
1,25(OH)_2_D_3_ (pg/ml)	0.098	0.027	0.064	0.153	0.070	0.116
VMR	−0.022	0.622	−0.026	0.567	−0.007	0.881
Urine						
25(OH)D (ng/g·Cre)^1^	0.031	0.493	0.050	0.262	0.048	0.289
3*-epi-*25(OH)D_3_ (ng/g·Cre)	−0.012	0.848	0.040	0.528	−0.040	0.523
24,25(OH)_2_D_3_ (ng/g·Cre)	−0.074	0.097	−0.058	0.192	−0.047	0.296
23,25(OH)_2_D_3_ (ng/g·Cre)	−0.038	0.392	−0.037	0.411	0.002	0.957
VMR	−0.141	0.001	−0.138	0.002	−0.140	0.002

Total P1NP, type I procollagen N-terminal propeptide; β-CTx, type I collagen cross-linked C-telopeptide; VMR, vitamin D metabolite ratio.

1 25(OH)D is a summation of 25(OH)D_3_ and 25(OH)D_2_.

2 Spearman’s rank correlation coefficient, two-sided.

## Discussion

The present study investigated the utility of urinary vitamin D metabolites as non-invasive biomarkers of the vitamin D status and obtained two principal results. Urinary 24,25(OH)_2_D_3_ and 23,25(OH)_2_D_3_ strongly correlated with serum 25(OH)D, indicating that urinary levels reflect the circulating vitamin D status. Furthermore, urinary metabolites and the VMR showed stronger inverse correlations with PTH levels than their serum counterparts. These results suggest the potential of urinary markers, particularly 24,25(OH)_2_D_3_ and the VMR, as reliable and superior alternatives to serum markers for assessing vitamin D sufficiency.

The present results on serum were consistent with previous studies showing that 24,25(OH)_2_D_3_ correlated with 25(OH)D [27] and inversely with PTH [3,4,26]. The inverse relationship between 24,25(OH)_2_D_3_ and PTH may be explained by the PTH-mediated suppression of CYP24A1, the enzyme responsible for converting 25(OH)D into 24,25(OH)_2_D_3_ [28]. The VMR also negatively correlated with PTH, which is consistent with previous findings [4], further supporting its utility as a functional marker of the vitamin D status. These results strengthen the view that 24,25(OH)_2_D_3_ and the VMR serve as informative markers of vitamin D metabolism and calcium homeostasis.

In contrast, serum 1,25(OH)_2_D_3_ did not correlate with 25(OH)D or PTH, which may be attributed to its complex, multilayered regulation. While PTH stimulates the production of 1,25(OH)_2_D_3_ via the up-regulation of CYP27B1 [29], FGF23 suppresses its synthesis and promotes its degradation by increasing CYP24A1 expression [29,30]. Furthermore, 1,25(OH)_2_D_3_ itself induces FGF23 [31] and suppresses the transcription of both CYP27B1 and CYP24A1 via feedback regulation [5,6,32]. These opposing regulatory pathways may obscure simple linear associations with other vitamin D-related parameters.

A notable result in the present study was the high percentage (33.6%) of participants with a low VMR (<4%), which contrasts sharply with European cohorts. For example, only 7.0% of community-dwelling older adults in Belgium [4] and 4.6% of healthy Austrian adults [3] had similarly low VMR values. Hsu et al*.* previously reported that White individuals had higher 24,25(OH)_2_D_3_ to 25(OH)D ratios, suggesting that genetic or ethnic differences in vitamin D metabolism affect the VMR [33]. Besides genetic factors, differences in sunlight exposure, dietary patterns, and the low use of vitamin D supplements in Japan may also be contributing factors.

Despite the clear correlations observed between urinary and serum vitamin D metabolites, the mechanisms underlying the relationship between urinary metabolites and PTH remain unclear. We showed that urinary 24,25(OH)_2_D_3_ and VMR not only correlated with serum 25(OH)D, but also showed stronger inverse relationships with PTH. One potential explanation is that urinary vitamin D metabolites are affected less by short-term hormonal regulation and are more reflective of cumulative vitamin D turnover. Vitamin D metabolites are freely filtered by the glomerulus and reabsorbed in the proximal tubule via megalin-mediated endocytosis [34]. Differences in the affinity of megalin for 24,25(OH)_2_D_3_ versus 25(OH)D may affect excretion rates, making the urinary profile more sensitive to upstream metabolic changes, including PTH activity.

In addition to its relationship with PTH, urinary VMR negatively correlated with bone turnover markers, suggesting its broader relevance in bone metabolism monitoring. This result highlights the potential utility of urinary VMR not only in assessing the vitamin D status, but also in providing indirect information about skeletal dynamics. Since bone remodeling is tightly regulated by vitamin D and PTH, the observed relationship between VMR and bone turnover may reflect an integrative marker of bone metabolic activity. Further studies are needed to confirm this relationship and examine its clinical applicability in bone health monitoring.

A major advantage of a urinary measurement is its non-invasive nature. Urine collection is simple, painless, and amenable to self-sampling, making it suitable for large-scale screening, particularly among populations for whom venipuncture is challenging (e.g. children, pregnant women, and the elderly). While urine-based assays require additional pre-treatments, including the hydrolysis of glucuronide conjugates, the present results suggest that the information obtained—particularly regarding PTH-related vitamin D metabolism—may exceed that from a serum analysis in some contexts.

The present study has several limitations. The effectiveness of hydrolysis using β-glucuronidase was not confirmed. Since unconjugated vitamin D metabolites are generally not detected in urine [9], incomplete hydrolysis may result in an underestimation. Enzyme activity varies with pH, and urine pH may fluctuate widely among individuals. A drug metabolism study reported that β-glucuronidase activity significantly varied depending on pH [35]. Therefore, the optimization of hydrolysis conditions and validation protocols are warranted. The satisfactory spike-and-recovery results obtained in the present study suggest that the sample preparation procedure for removing interfering substances and liquid chromatography gradient conditions effectively reduced matrix-related interference. However, since the spike-and-recovery tests were conducted using pooled urine samples, inter-individual variability in the urinary matrix composition may have been averaged out. Therefore, matrix effects and ion suppression in individual urine samples may not have been fully captured. Further validation using urine samples obtained from multiple individuals is warranted. Furthermore, information on vitamin D supplementation was unavailable due to limitations in the health check-up questionnaire. A previous study showed that urinary 25(OH)D_3_ and 24,25(OH)_2_D_3_ both increased after vitamin D supplementation [17]; however, the rate of the increase may differ and affect the VMR. In addition, diurnal variations in urinary vitamin D metabolites remain unexamined. Rejnmark et al. reported circadian fluctuations in serum 1,25(OH)_2_D of ±10% over 24 h [36], suggesting that spot urine samples are affected by the collection time. We are currently investigating this aspect in a separate cohort. Another limitation is that while we adjusted for creatinine, this approach may be inappropriate for populations with impaired renal function, in whom both vitamin D and creatinine excretion are affected. In addition, inter-individual differences in renal function may affect urinary vitamin D metabolite excretion through changes in glomerular filtration, protein handling, and tubular reabsorption. Approximately 99% of circulating vitamin D metabolites are bound to vitamin D-binding protein or albumin [37]. Therefore, renal disorders that affect the filtration, reabsorption, or urinary loss of these carrier proteins, such as chronic kidney disease and nephrotic syndrome, may affect urinary vitamin D metabolite levels. Serum 25(OH)D concentrations are frequently reduced in patients with chronic kidney disease [38], further complicating the interpretation of urinary vitamin D metabolite profiles. Although few studies have investigated urinary vitamin D metabolites in these populations, these factors need to be considered when evaluating urinary metabolite profiles. Our participants were generally healthy; therefore, future studies need to evaluate these markers in clinical populations.

In conclusion, urinary vitamin D metabolites—particularly 24,25(OH)_2_D_3_, 23,25(OH)_2_D_3_, and the VMR—were strongly associated with both serum 25(OH)D and PTH, and may offer a practical, non-invasive alternative for evaluating the vitamin D status. These results support the further development of urine-based assays and highlight their potential value in clinical and public health settings.

## Supplementary Material

Supplementary Figures S1-S4 and Tables S1-S2

## Data Availability

All data supporting the results of this study are available from the corresponding author (Nau Ishimine, now3014@shinshu-u.ac.jp) upon reasonable request.

## Abbreviations

β-CTx: type I collagen cross-linked C-telopeptide
γ-GT: gamma-glutamyl transferase
1,25(OH)_2_D: 1,25-dihydroxyvitamin D
23,25(OH)_2_D_3_: 23,25-dihydroxyvitamin D_3_
24,25(OH)_2_D_3_: 24,25-dihydroxyvitamin D_3_
25(OH)D: 25-hydroxyvitamin D
25(OH)D_2_: 25-hydroxyvitamin D_2_
25(OH)D_3_: 25-hydroxyvitamin D_3_
3-*epi*-25(OH)D_3_: 3-*epi*-25-hydroxyvitamin D_3_
ALT: alanine aminotransferase
AST: aspartate aminotransferase
CV: coefficient of variation
CYP: cytochrome P450
DAP-PA: 14-(4-(dimethylamino)phenyl)-9-phenyl-9,10-dihydro-9,10-[1,2]epitriazoloanthracene-13,15-dione
DAPTAD: 4-(4′-dimethylaminophenyl)-1,2,4-triazolidine-3,5-dione
ESI: ectrospray ionization
FGF23: fibroblast growth factor 23
THF: tetrahydrofuran
IQR: interquartile range
IS: internal standard
LC–MS/MS: liquid chromatography-tandem mass spectrometry
PLC: preparative-TLC
PTH: parathyroid hormone
SLE: solid–liquid extraction
SRM: selected reaction monitoring
total P1NP: type I procollagen N-terminal propeptide
VMR: vitamin D metabolite ratio
