# Supplementary material for: Evaluation of correlations between serum and urinary vitamin D metabolites using LC–MS/MS
Source: Biosci Rep. 2026 Jul 1;46(7):BSR20260223. doi: 10.1042/BSR20260223 (PMC13329322; doi:10.1042/BSR20260223)
Supplement: Supplementary Figures S1-S4 and Tables S1-S2 [file BSR-2026-0223_supp.pdf]

Supplementary Data

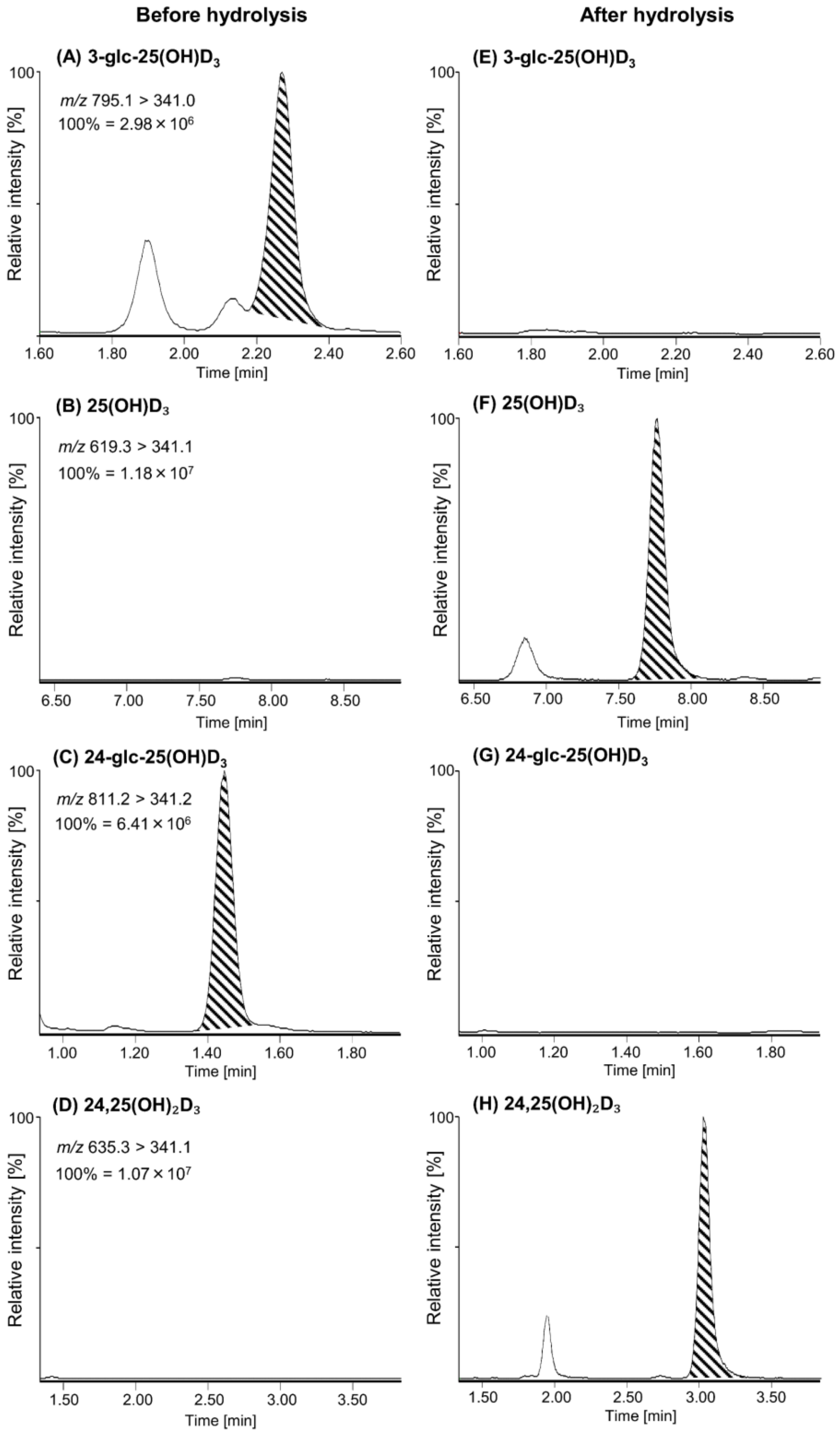

Supplementary Figure S1. SRM chromatograms of glucuronidated and non-conjugated vitamin D metabolites in spiked urine samples before and after enzymatic hydrolysis

Selected reaction monitoring (SRM) chromatograms of vitamin D metabolites derivatized with DAP-PA,

obtained from pooled human urine samples spiked with synthesized glucuronidated standards (10 ng/mL each), are shown. before (left column, **A**-**D**) and after (right column, **E**-**H**) enzymatic hydrolysis with β-glucuronidase.

(A) 3-glc-25(OH)D_3 detected before hydrolysis.

(B) 25(OH)D_3 detected before hydrolysis.

(C) 24-glc-25(OH)D_3 detected before hydrolysis.

(D) 24,25$(OH)_2D_3$ detected before hydrolysis.

(E) 3-glc-25(OH)D_3 after hydrolysis.

(F) 25(OH)D_3 after hydrolysis.

(G) 24-glc-25(OH)D_3 after hydrolysis.

(H) 24,25$(OH)_2D_3$ after hydrolysis.

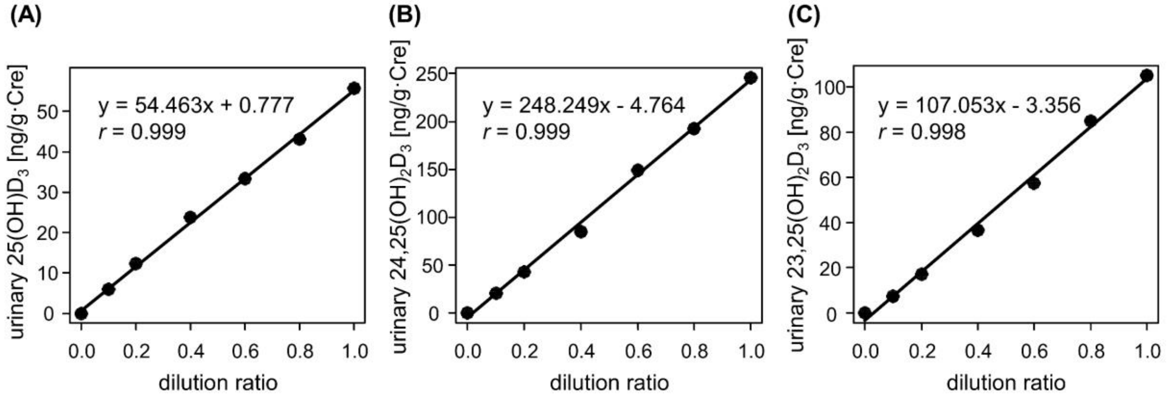

Supplementary Figure S2. The linearity test for urinary vitamin D metabolites

The dilution linearities of urinary **(A)** 25(OH)D_3, **(B)** 24,25$(OH)_2D_3$, and **(C)** 23,25$(OH)_2D_3$ were assessed, and all achieved high linearity.

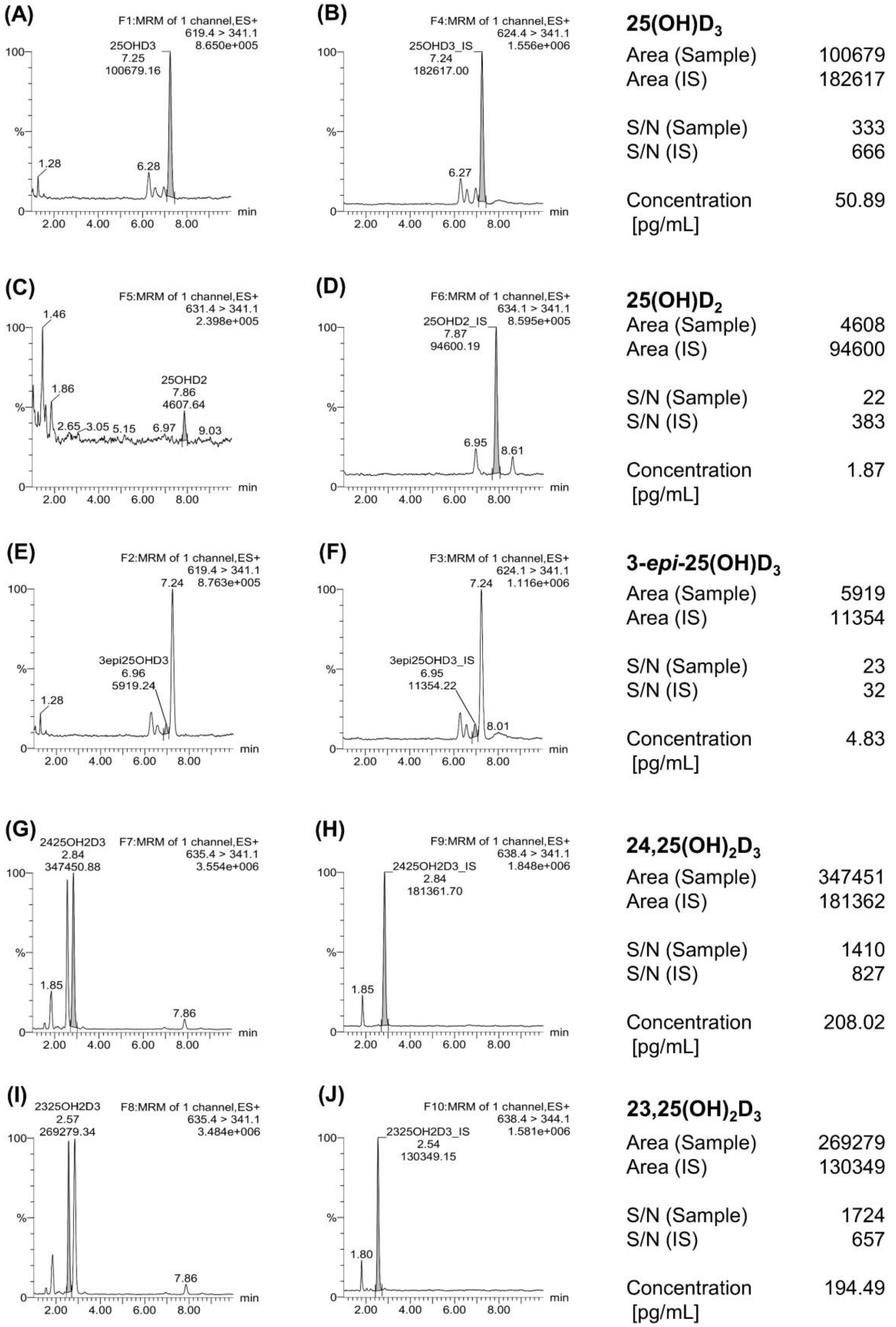

Supplementary Figure S3. Representative chromatograms and raw data obtained from a urine sample

Representative chromatograms and raw data obtained from a urine sample are shown. IS, internal standard; S/N, signal-to-noise ratio.

(A) urinary 25(OH)D_3

(B) urinary 25(OH)D_3-IS

(C) urinary 25(OH)D_2

(D) urinary 25(OH)D_2-IS

(E) urinary 3-*epi*-25(OH)D_3

(F) urinary 3-*epi*-25(OH)D_3-IS

(G) urinary 24,25$(OH)_2D_3$

(H) urinary 24,25$(OH)_2D_3$-IS

(I) urinary 23,25$(OH)_2D_3$

(J) urinary 23,25$(OH)_2D_3$-IS

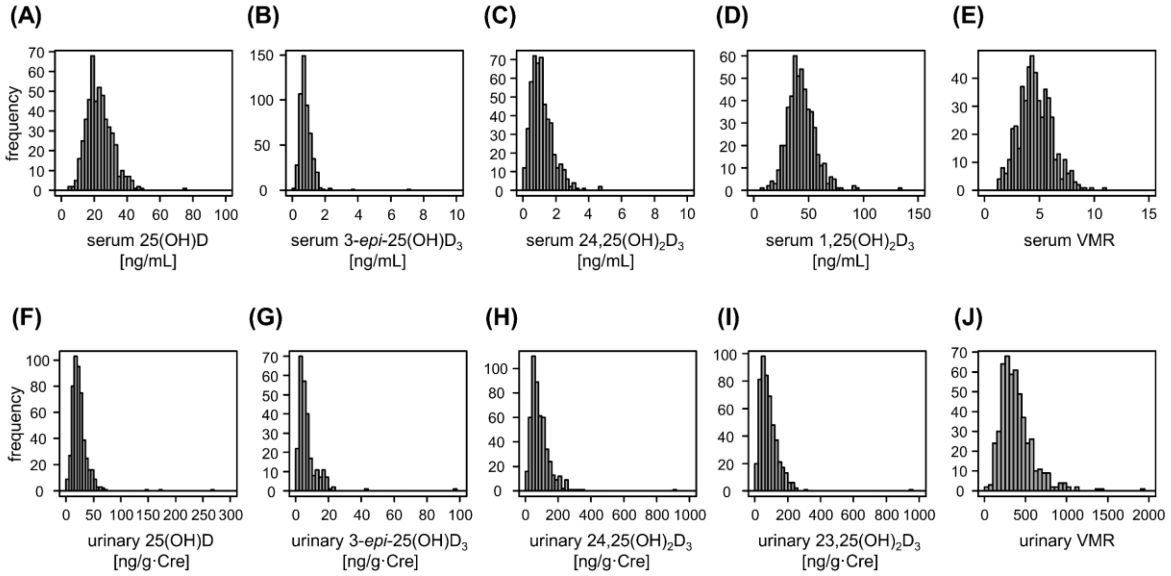

Supplementary Figure S4. The profile of vitamin D metabolites among a seemingly healthy population in Japan

The distributions of serum (top row, **A**-**E**) and urinary (bottom row, **F**-**J**) vitamin D metabolites and the VMR (vitamin D metabolite ratio) among seemingly healthy employees at Shinshu University Hospital, Japan are shown.

(A) serum 25(OH)D

(B) serum 3-*epi*-25(OH)D_3

(C) serum 24,25$(OH)_2D_3$

(D) serum 1,25$(OH)_2D_3$

(E) serum VMR

(F) urinary 25(OH)D

(G) urinary 3-*epi*-25(OH)D_3

(H) urinary 24,25$(OH)_2D_3$

(I) urinary 23,25$(OH)_2D_3$

(J) urinary VMR

Abbreviations

25(OH)D, 25-hydroxyvitamin D; 25$(OH)D_3$, 25-hydroxyvitamin D_3; 25$(OH)D_2$, 25-hydroxyvitamin D_2; 3-*epi*-25$(OH)D_3$, 3-*epi*-25-hydroxyvitamin D_3; 24,25$(OH)_2D_3$, 24,25-dihydroxyvitamin D_3; 1,25$(OH)_2D_3$, 1,25-dihydroxyvitamin D_3; 23,25$(OH)_2D_3$, 23,25-dihydroxyvitamin D_3; VMR, the vitamin D metabolite ratio.

Supplementary Data

Supplementary Table S1. Within-run and between-run precision of the LC-MS/MS assay for serum vitamin D metabolites

	$25(OH)D_3$		$25(OH)D_2$		3-*epi*-$25(OH)D_3$		$24,25(OH)_2D_3$	
	Mean ± SD [ng/mL]	CV [%]	Mean ± SD [ng/mL]	CV [%]	Mean ± SD [ng/mL]	CV [%]	Mean ± SD [ng/mL]	CV [%]
**Within-run (n = 5)**								
Low	7.82 ± 0.06	0.8	0.23 ± 0.01	5.2	0.32 ± 0.01	2.0	0.08 ± 0.002	2.6
High	25.81 ± 0.23	0.9	1.73 ± 0.02	1.0	1.42 ± 0.03	1.9	0.49 ± 0.004	0.8
**Between-run (n = 5)**								
Low	8.54 ± 0.50	5.8	0.23 ± 0.01	6.0	0.36 ± 0.02	6.8	0.08 ± 0.003	4.1
High	29.61 ± 2.21	7.4	1.88 ± 0.12	6.1	1.64 ± 0.13	7.7	0.56 ± 0.05	8.2

SD, standard deviation; CV, coefficient of variation.

Supplementary Table S2. Within-run and between-run precision of the LC-MS/MS assay for urinary vitamin D metabolites

	$25(OH)D_3$		$25(OH)D_2$		3-*epi*-$25(OH)D_3$		$24,25(OH)_2D_3$		$23,25(OH)_2D_3$	
	Mean ± SD [pg/mL]	CV [%]	Mean ± SD [pg/mL]	CV [%]	Mean ± SD [pg/mL]	CV [%]	Mean ± SD [pg/mL]	CV [%]	Mean ± SD [pg/mL]	CV [%]
Within-run (n = 4)	25.64 ± 0.52	2.0	N.D.		3.06 ± 0.95	31.0	48.61 ± 1.86	3.8	52.56 ± 2.36	4.5
Between-run	(n = 6)		(n = 1)		(n = 4)		(n = 6)		(n = 6)	
	25.89 ± 2.23	8.6	0.271	N.A.	5.71 ± 2.37	41.5	47.62 ± 4.60	9.7	51.12 ± 1.40	2.7

N.D., not detected; N.A., not available; SD, standard deviation; CV, coefficient of variation.

Supplementary Table S3. Spike-and-recovery test of urinary vitamin D metabolites

Added substance	Actual spiked concentration [pg/mL]	Obtained concentration on average [pg/mL]	CV [%]	Retrieved concentration [pg/mL]	Recovery [%]
		$25(OH)D_3$ (n = 4)			
+ distilled water	0.00	44.08	2.5	N.A.	N.A.
+ 100 pg/mL of calibrator	20.00	65.53	2.0	21.45	107.3
+ 800 pg/mL of calibrator	160.00	208.33	5.7	164.25	102.7
		$24,25(OH)_2D_3$ (n = 4)			
+ distilled water	0.00	187.18	1.7	N.A.	N.A.
+ 100 pg/mL of calibrator	20.00	209.65	3.7	22.48	112.4
+ 800 pg/mL of calibrator	160.00	346.35	3.2	159.18	99.5
		$23,25(OH)_2D_3$ (n = 4)			
+ distilled water	0.00	80.35	1.4	N.A.	N.A.
+ 100 pg/mL of calibrator	20.00	100.53	2.8	20.18	100.9
+ 800 pg/mL of calibrator	160.00	221.28	0.9	140.93	88.1

N.A., not available; CV, coefficient of variation.
